# Ethical Norms, Challenges, and Associated Factors in Telemental Health: Perspectives from Psychiatric and Psychological Professionals in China

**DOI:** 10.3390/healthcare14111472

**Published:** 2026-05-26

**Authors:** Xinyi Chang, Xinyue Hu, Yang Shao, Yi Qiao

**Affiliations:** 1 Shanghai Mental Health Center, Shanghai Jiao Tong University School of Medicine, Shanghai 200030, China; changxinyi01@sjtu.edu.cn; 2 Student Counseling and Mental Health Center, Jiaxing Vocational & Technical College, Jiaxing 314036, China; huxinyue@jxvtc.edu.cn

**Keywords:** telemental health, differences between psychiatrists and psychologists, ethical norms

## Abstract

**Background:** With the rising demand for psychiatric mental health services and the development of online technology, telemental health services are gaining popularity. Psychiatrists and psychologists differ significantly in service patterns and ethical models. This study investigated their ethical patterns and used the technology acceptance model (TAM) to explore how professionals’ attitudes influence their ethical and regulatory use of telemental health services. **Methods:** The online survey included their basic information, telemental health service patterns, attitudes toward telemental health services, and ethical norms. This cross-sectional online survey was conducted among psychiatrists and psychologists in China between April and October 2022. Of the 1071 respondents in the parent survey, 690 professionals who reported using telemental health services were included in the present subgroup analysis. **Results:** In some instances, practitioners offering telemental health services may not adhere to ethical standards, particularly in the case of psychologists. A significant proportion of respondents expressed concerns including potential emergencies, technical issues, and security, suggesting the need for a re-evaluation of the ethical framework. The TAM showed higher behavioral intention was associated with lower ethical compliance scores. Conversely, elevated subjective norms and perceived behavioral control have the potential to encourage ethical compliance. **Conclusions:** Telemental health services are widely used in China, but important gaps remain in ethical compliance and regulatory implementation. Future efforts should focus on strengthening professional training, improving platform security and emergency response procedures, and developing clearer institutional and professional guidelines for ethical telemental health practice.

## 1. Introduction

According to the World Mental Health Report 2022, 71% of people with mental problems do not have access to mental health services, a situation worsened by the COVID-19 pandemic. In response, telemental health services have developed. These services use telecommunication or videoconferencing technology to provide mental health services, including via video calls, smartphone apps and email [1]. Telemental health services utilize limited resources to reach more people [2], improve patient compliance [3], enable increased coverage [4], reduce patient financial burden [5], and improve continuity of care for patients [6,7].

Global adoption of telemental health surged during the pandemic [8,9]. In the U.S., nearly all therapists transitioned to telemental health platforms [10,11], while usage in the Asia-Pacific region more than doubled during the pandemic [12]. Similarly, 58% of Spanish-speaking therapists reported using telemental health services, and this number rose to 84% during the pandemic [13]. Even after the pandemic, countries like France continued to expand their use of such services, reflecting growing acceptance and integration [14]. The COVID-19 pandemic boosted the popularity of telemental health services and increased user acceptance. Evidence from other health-related digital settings also suggests that the adoption of new technologies is shaped not only by technical feasibility, but also by perceived usefulness, ease of use, and appropriate implementation. For example, CBCT-based e-learning in dental anatomy education improved learning outcomes and student satisfaction, highlighting the importance of structured support when introducing digital tools [15]. This parallel is relevant to telemental health, where adoption may likewise be associated with whether professionals perceive remote technologies as useful, manageable, and adequately supported by training and ethical guidance.

However, as a new form of service, telemental health raises new concerns that differ from traditional settings. Challenges include the relationship between counselor and client, the service process, projection in the network, the phenomenon of empathy, interview techniques, and psychological intervention methods [16,17,18]. More critically, new ethical issues have emerged, such as privacy issues, inadequate content of informed consent [19], and lack of targeted training [20].

In recent years, telemental health service has undergone rapid development in China, being particularly accelerated during the COVID-19 pandemic [21]. It has become increasingly diverse in terms of service modalities, target populations, and types of interventions, indicating substantial potential for future development [22]. Research has shown that practitioners from different professional backgrounds—such as psychiatrists and psychological counselors—exhibit significant differences in ethical standards [23]. However, what remains insufficiently understood in the Chinese context is not only the overall expansion of telemental health, but also how it is actually practiced in routine settings, how closely current practice aligns with ethical norms, and which attitudinal factors are associated with more standardized use. Psychiatrists and psychologists are a particularly important comparison in this context because they represent two major groups providing telemental health services, yet they differ in training background, institutional setting, service tasks, and professional ethical traditions. These differences may be associated with distinct patterns of platform use, documentation, emergency management, and ethical risk. In order to clearly define the current ethical status of telemental health services in China, this study not only focused on the current situation, but also examined which factors were associated with more standardized and ethical telemental health practice.

This study applied the technology acceptance model (TAM) to examine how professionals’ attitudes are associated with both the use and ethical application of telemental health services. The TAM has been widely applied to investigate the adoption of new technologies in psychiatric services, particularly within the context of telemental health [24,25]. In the present study, the TAM is used not only as a framework for technology uptake, but also as a way to understand ethical practice in a technology-mediated setting. In telemental health, ethical conduct is enacted through concrete decisions about whether to use telemental health services, which platforms to use, whether informed consent and privacy procedures can be implemented effectively, and whether psychiatrists feel able to manage crisis situations online. Attitudes toward the use of telemental health services, subjective norms, perceived usefulness, perceived ease of use, behavioral intentions, and percentage of current telemental health service use were significantly positively correlated [26]. Subjective norms significantly predicted perceived usefulness and perceived ease of use, which in turn predicted actual use of telemental health services [27].

While little research has addressed the relationship between professionals’ attitudes and ethical normativity, this study examined whether attitude-related factors in the TAM are associated with ethical standards in the delivery of telemental health services. Accordingly, this study aimed to describe current telemental health practice and ethical status among mental health professionals in China and to examine whether TAM-related factors were associated with telemental health utilization and ethical compliance. We expected all TAM constructs to be positively associated with utilization, whereas attitude, perceived usefulness, perceived ease of use, subjective norms, and perceived behavioral control were expected to be positively associated with ethical norms, with the strongest associations expected for subjective norms and perceived behavioral control. The association between behavioral intention and ethical norms was examined exploratorily. We also expected current practice in China to vary across professional groups and not always fully meet existing ethical expectations.

## 2. Materials and Methods

### 2.1. Participants and Procedure

Our study was a subgroup analysis of data from an online survey series using a structured questionnaire between April and October 2022. Ethics approval was obtained from the Shanghai Mental Health Center (IORG0002202, FWA00003065). Participants were psychiatric and psychological service professionals in China. Under China’s Mental Health Law [28], psychotherapists may treat mental disorder patients in medical settings, whereas psychological counselors only offer counseling in non-medical venues like communities or schools [29]. Both are termed “psychologists,” but neither can diagnose mental disorders or prescribe medication. In contrast, psychiatrists are medical doctors who work in hospitals or psychiatric clinics and have the authority to diagnose mental disorders.

Sample data were collected through an online questionnaire survey. The questionnaire was developed on the Wenjuanxing platform (www.wjx.cn) and disseminated through WeChat groups to members of the Psychiatric Branch of the Chinese Medical Doctor Association and the Chinese Mental Health Association. To reduce topic-related self-selection, the survey was posted under the neutral title “Survey on Telemental Health Services.” Members of these associations were invited to complete the survey and to share the survey link with other psychiatrists and psychological professionals in their networks. A quality-control item was embedded in the questionnaire to ensure data quality. Questionnaires with an incorrect response to the quality-control item or with obvious logical inconsistencies were excluded as invalid. In total, 1249 mental health professionals completed the online questionnaire and 1071 valid questionnaires were retained after data cleaning. For the present sub-analysis, 690 respondents who reported using telemental health services were included in the final analysis. Other findings related to patient-targeted Googling (PTG) were reported by Feng [23].

### 2.2. Questionnaire Content

The questionnaire consisted of four sections: demographics, service patterns, attitudes, and ethical norms (see Appendix A for details).

#### 2.2.1. Basic Information

The demographic section was based on a self-designed questionnaire, including the age, gender, professional background in mental health services (psychiatrists/psychologist), highest degree (PhD/master’s/undergraduate/other), and main workplace of the surveyed professionals.

#### 2.2.2. Telemental Health Service Patterns

The service pattern questionnaire focused on the respondents’ actual mode, including the platform used to provide telemental health services, the content of the services provided, the venues where the services are provided, the fees charged, the informed consent, the technical safeguards, and the relevant training, etc. For the regression analyses, the outcome variable was participants’ self-reported average number of hours per week spent providing telemental health services.

#### 2.2.3. Attitudes Toward Telemental Health Services

The Attitudinal Perceptions Questionnaire was adapted from Chau and Hu [25], and included 21 items across six dimensions: attitude (ATT), subjective norms (SN), perceived behavioral control (PBC), perceived usefulness (PU), perceived ease of use (PEOU), and behavioral intention (BI). Items were rated on a 7-point Likert scale, with 10 items reverse-scored. Total scores ranged from 21 to 147. With permission from the original authors, the Attitudinal Perceptions Questionnaire was translated and adapted. Two researchers independently translated the English version into Chinese, followed by expert review and back-translation. A pilot test on 22 mental health providers confirmed clarity. Test–retest reliability for the total score was good (Pearson’s r = 0.848, *p* < 0.01), and internal consistency was excellent (Cronbach’s α = 0.918). In the present sample, Cronbach’s α coefficients for the six subscales ranged from 0.561 to 0.768. Based on previous studies, items from the same dimension were randomized in the questionnaire to reduce response bias.

#### 2.2.4. Ethical Norms

The ethics questionnaire was based on the Expert Consensus on the Management Norms and Ethical Guiding Principles for Telepsychological Services [30], with a total of 19 items. It included the signing of the service agreement, matters to be informed of in advance, the choice of the service environment, the evaluation and adjustment of the service program, and the preservation and retention period of service records. Responses were measured on a 7-point Likert scale, with 5 items reverse-scored. The total score ranged from 19 to 133, with higher scores indicating greater ethical compliance. The questionnaire was pilot-tested in 22 mental health service providers to confirm that the wording was understandable and did not cause difficulty in comprehension. One week later, the questionnaire was re-administered to the same participants. Test–retest reliability for the total score was acceptable (Pearson’s r = 0.788, *p* < 0.001), and internal consistency was good (Cronbach’s α = 0.839).

For the regression analyses, the total ethics questionnaire score was used as the ethical outcome variable. In the present sample, the questionnaire showed low internal consistency (Cronbach’s α = 0.518). Given that the instrument encompasses several conceptually distinct domains of ethical practice, this coefficient may partly reflect heterogeneity in item content. Nevertheless, because the aim of the present study was to assess overall ethical compliance rather than domain-specific differences in ethical behavior, the total score was retained as a single outcome and interpreted with caution.

### 2.3. Data Analysis

Data were analyzed using SPSS 23.0. Chi-squared and *t*-tests compared differences in the cognitive attitudes toward telemental health services among service providers with different professional backgrounds. Multiple regression was used to examine factors associated with telemental health services utilization and ethical compliance. Collinearity diagnostics indicated no problematic multicollinearity among predictors (tolerance = 0.380–0.670; VIF = 1.493–2.629). All questionnaire items were mandatory on the online survey platform; therefore, the retained questionnaires contained no missing data.

## 3. Results

### 3.1. Demographics of Participants

A total of 690 respondents used telemental health services in their work, including 218 psychiatrists and 472 psychologists. The average age of participants was 40.47 years. Compared with psychologists, psychiatrists generally had more years of practice, were more likely to hold doctoral degrees, and were much more concentrated in public institutions. By contrast, psychologists were more likely to hold master’s degrees and were more evenly distributed across public and private settings. The sample was predominantly female overall, although male representation was higher among psychiatrists. Details are presented in Table 1.

### 3.2. Basic Service Pattern

The main service patterns are summarized in Table 2. Video, free hotlines, and telephone were the most commonly used modalities. Psychiatrists more often used text-based, telephone, and follow-up diagnostic services, whereas psychologists were more likely to use video-based services. Adults and adolescents were the main service users, although psychiatrists reported relatively more interactions with children and older adults. Most respondents indicated that telemental health services were priced similarly to or lower than on-site services. Treatment orientation also differed between groups, with psychiatrists more often using CBT and psychologists more often using psychoanalytic therapy.

### 3.3. Ethical Service Details

Ethical service patterns differed between psychiatrists and psychologists, as shown in Table 3. Psychiatrists more often relied on institution-based or professional platforms and institutional record systems, whereas psychologists more often used general communication tools, private spaces, and self-maintained documentation. These patterns suggest different ethical risk profiles across the two groups. Psychiatrists were less likely than psychologists to report receiving relevant training, whereas psychologists, particularly those in private institutions, more often emphasized users’ right to terminate services.

Nearly all respondents expressed at least one concern about telemental health services. The most common concerns involved emergencies, technical issues, data security, and relationship-building. Emergency response capacity appeared limited, as relatively few respondents reported access to location information, institutional coordination with safety authorities, or formal transfer arrangements, and many indicated that they could only recommend offline care. Concerns also varied across professional and demographic groups, with psychiatrists more often reporting concerns related to standardization, confidentiality, training, and research evidence, and psychologists more often reporting technical concerns. Detailed results are presented in Table 4.

Regarding professional competency and responsibility, substantial proportions of respondents reported limited training in the practical, legal, and ethical aspects of telemental health services, as well as incomplete knowledge of relevant laws, regulations, and professional standards. Institutional regulation also remained limited, with fewer than half reporting that their organizations had specific rules for telemental health services. Although many respondents informed users about benefits/risks and data security, fewer addressed data storage, alternative service options, or users’ right to terminate services. Detailed results are presented in Table 5.

### 3.4. Factors Associated with Telemental Health Utilization and Ethical Compliance

To extend the descriptive findings above, two multiple linear regression analyses were conducted to examine factors associated with telemental health utilization and ethical compliance. The first model showed that profession (B = 19.748, *p* < 0.001) and workplace (B = −20.338, *p* < 0.001) were significant factors, with psychologists and those in private institutions using telemental health services more frequently. Behavioral intention was also significantly associated with greater use of telemental health services (B = 1.314, *p* < 0.001). Details are presented in Table 6.

In the second model, neither profession nor workplace was significantly associated with ethical compliance. Subjective norms (B = 0.401, *p* = 0.018) and perceived behavioral control (B = 0.427, *p* < 0.001) were positively associated with ethical compliance, while behavioral intention (B = −0.545, *p* < 0.001) was negatively associated with ethical compliance. Details are presented in Table 7.

## 4. Discussion

This study explored the ethical model and potential influencing factors of telemental health services in China. In summary, ethical considerations remain significantly deficient in process confidentiality, informed consent, practitioner training, legal formulation, etc. Distinct groups, especially psychiatrists in public organizations and psychologists in private organizations, face ethical risks at various critical points.

### 4.1. Group Differences in Telemental Health Ethics

Psychiatrists tend to use institutional platforms and specialized platforms, which typically offer more comprehensive encryption, identity verification, and data storage management functions, better meeting the ethical requirements for confidentiality and data security. In contrast, the general business software and social media tools commonly employed by psychologists, although flexible and convenient, lack security features specifically designed for mental health services, posing significant risks of information leakage and breaches of data privacy. Secondly, a considerable proportion of both psychiatrists and psychologists deliver services from private spaces, resulting in an elevated risk of interruption and typically providing inferior soundproofing and privacy protection relative to dedicated institutional spaces. These environmental factors pose a risk to clients’ trust and consultation quality. Furthermore, the institutional electronic records utilized by psychiatrists facilitate compliance with legal and ethical traceability requirements and reduce the risk of loss or tampering, whereas the self-maintained records employed by psychologists, while flexible, are more likely to lack safeguards about data security, retention periods, and access permissions. Collectively, the platforms’ compliance, the confidentiality of the service environment, and the institutionalization of documentation processes are key ethical risk factors.

The ethical risk points differ by professional background, reflecting variations in institutional support and service goals. Most psychiatrists are affiliated with public medical institutions, benefiting from well-developed technical infrastructure and institutional support. However, they exhibit lower participation rates in ethics training compared to psychologists, potentially attributable to medical training programs placing greater emphasis on clinical skills and medical record standards. Consequently, psychiatrists’ ethical risks are more concentrated in the realm of relatively insufficient awareness of detailed ethical guidelines. Psychologists, in contrast, are more widely distributed across sectors, with a high proportion working in private institutions. Private institutions often prioritize service efficiency and user experience, which may be associated with insufficient investment in emergency preparedness and compliance procedures, increasing exposure to technical risks such as information security vulnerabilities, breaches of privacy, and inadequate documentation.

Overall, our findings are consistent with those of studies in other cultures [31,32]. Consistent with other worldwide studies [33], differences in the ethical concerns expressed by various groups highlight distinct training needs and priorities for institutional support. Psychiatrists may require more detailed operational guidelines and standardized tools tailored to ethical practice in telemental health contexts, whereas psychologists would derive greater benefit from training and resources focused on technical security and data protection. For public institutions, priority should be given to updating standardized procedures and enhancing the adaptation of ethical guidelines for service contexts. For private institutions, efforts should focus on enhancing technical security capabilities, establishing emergency response mechanisms, and implementing robust documentation systems.

### 4.2. Determinants of Ethical Practice in Telemental Health

This study found that in addition to the occupational background and work location, behavioral intention was most strongly associated with the percentage of service use, though factors such as perceived usefulness and perceived ease of use, identified in previous studies, were also significant [26]. This finding is consistent with the theoretical framework of the TAM, in which behavioral intention is strongly associated with perceived usefulness [34]. One possible explanation is that high behavioral motivation may be associated with greater professionals’ telepresence (i.e., to forget working remotely and to feel present with the client) [35] and in turn they have a more positive attitude toward telemental health services [31].

Regarding ethical norms, subjective norms and perceived behavioral control had a significant positive effect on ethical norms scores, while behavioral intention had a significant negative effect. These results partially support the theory of the TAM and reveal the complex relationship between behavioral intentions and ethical norms. Subjective norms reflect the influence of social pressures perceived from ethics committees, supervisors, clients, and colleagues. The positive effect of subjective norms suggests that stronger perceived social pressures may be associated with greater adherence to ethical norms. Perceived behavioral control reflects one’s ability to telemental health services. The higher the perceived behavioral control, the more confident professionals are in following the ethical norms. However, the negative effect of behavioral intention on ethical norms may reflect the ethical challenges of telemental health services during rapid development. This suggests that, even when professionals strongly endorse the value and convenience of telemental health services, high behavioral motivation does not necessarily translate into ethical self-regulation. One possible explanation is that professionals with strong motivation to provide telemental health may also operate under productivity pressures, financial incentives, or organizational environments in which service expansion is prioritized over rigorous ethics protocols. In such contexts, enthusiasm for digital service delivery may increase the frequency and efficiency of online practice without necessarily strengthening ethical safeguards. A useful parallel can be drawn from other domains of digital health and education: just as the adoption of CBCT-based e-learning in dental anatomy required structured implementation and oversight to ensure that greater technological engagement was linked with better, rather than merely faster, learning [15], the expansion of telemental health should likewise be accompanied by safeguards ensuring that high enthusiasm and frequent use are aligned with, rather than in tension with, ethical compliance. Ethical compliance may be associated with a multifaceted set of factors, including not only knowledge and skills, but also institutional pressures and prevailing cultural attitudes.

### 4.3. Implications for Policy

This study identifies key ethical challenges in telemental health, informing both practice and policy. More specifically, the present findings point to two immediate priorities for practice and policy: updating national and institutional ethical guidelines specifically for telemental health services, and implementing structured, mandatory training on the legal, ethical, and technical aspects of telemental health, particularly crisis management and data security.

The professional training and ethical norms system for practitioners in China still needs to be improved. In 2018, the Chinese Psychological Association released its second code of ethics, applicable to telemental health. In 2019, Guan and Wang published an expert consensus on telepsychological standards and ethics. But neither of them has been updated in response to the rapid development of telemental health services in recent years [31,36,37]. International guidelines have likewise moved toward more explicit regulation of digital practice, as illustrated by the EFPA guide, first issued in 2006 and updated in 2023. Issues such as information storage patterns, alternative options and the right of the visitor to discontinue the service directly impact service quality. Updated guidance should also more clearly address emergency response pathways, platform security, documentation requirements, and the conditions under which telemental health services should be adjusted, transferred, or discontinued. Authorities and professional associations are recommended to systematically promote standardized training and institutional development, particularly supporting private institutions and beginning professionals. Training is a key factor in improving practitioners’ knowledge of telemental health services [31]. Such training should ideally be structured and mandatory, rather than optional or ad hoc, and should include practical competencies in online informed consent, data protection, crisis management, and the ethical and legal limits of telemental health services.

Professionals’ concerns, especially those related to technical issues and user trust, also need to be addressed through stronger platform security and clearer service protocols [38]. Use of low-tech formats enhanced accessibility for children and the elderly [39]. The lower engagement of children in telemental health services is consistent with U.S. findings [40,41], while the continued adult focus in contemporary research further highlights the need for age-appropriate service models [22]. Experiences from other areas of health and health education further suggest that advanced digital tools are most beneficial when they are embedded in a broader strategy of competency development and governance, rather than treated merely as a technological upgrade. For example, CBCT-based e-learning in dental anatomy emphasized “clear learning outcomes,” “continuous review and adjustment,” and “teacher supervision” [15], highlighting that effective digital practice depends not only on the technology itself but also on structured implementation and oversight.

### 4.4. Limitations

This study has several important limitations. First, the study design was cross-sectional. Therefore, the observed relationships reported in this study cannot be interpreted as directional or causal. In particular, we cannot determine whether the identified associations reflect temporal ordering, reverse causation, or other unmeasured institutional and professional factors. This limitation should be kept in mind when interpreting the findings and their implications. Second, the sample was drawn mainly from members of Chinese professional associations and may not adequately represent practitioners in remote or under-resourced settings, which limits the generalizability of the findings. In addition, because this sub-analysis was restricted to respondents who reported using telemental health services, the findings specifically reflect professionals with relevant experience and may not be generalizable to all mental health professionals in China. Third, the data relied on self-report, which may have introduced social desirability bias and recall bias, particularly for items concerning ethical conduct and service routines. Finally, this study focused on professionals’ reported practices and attitudes rather than patient outcomes or the comparative effectiveness of telemental health versus face-to-face care. Future longitudinal, multisite, and mixed-method studies are needed to clarify these relationships and to examine how regulatory, organizational, and training factors shape ethical practice over time.

## 5. Conclusions

Telemental health is already widely used among psychiatrists and psychologists in China, but important ethical and regulatory gaps remain. These findings underscore that the challenge is no longer whether telemental health is being used, but how it can be implemented more safely, consistently, and ethically across service settings. In particular, national and institutional ethical guidelines should be updated specifically for telemental health services, and structured, mandatory training should be implemented on the legal, ethical, and technical aspects of telemental health, especially crisis management and data security. More broadly, the development of telemental health should be embedded in a wider strategy of competency development, institutional governance, secure infrastructure, and ongoing quality assurance, rather than treated simply as a technological upgrade.

## Figures and Tables

**Table 1:** Demographics of participants to survey (*n* = 690).

	Total	Psychiatrists	Psychologists	χ^2^	t
	(*n* = 690)	(*n* = 218)	(*n* = 472)		
Age	40.47 ± 8.765	42.15 ± 8.004	39.70 ± 8.998		3.434 **
Service Years	8.40 ± 6.145	11.48 ± 6.878	6.98 ± 5.198		9.512 **
Gender				30.606 ***	
Male	206	96	110		
	(29.9%)	(44.0%)	(23.3%)		
Female	484	122	362		
	(70.1%)	(56.0%)	(76.7%)		
Highest Education				49.780 **	
PhD	52	35	17		
	(7.5%)	(16.1%)	(3.6%)		
Master’s Degree	264	56	208		
	(38.3%)	(25.7%)	(44.1%)		
Bachelor’s Degree	351	124	227		
	(50.9%)	(56.9%)	(48.1%)		
Other	23	3	20		
	(3.3%)	(1.4%)	(4.2%)		
Main Workplace				171.875 ***	
Public Institution	395	204	191		
	(57.2%)	(93.6%)	(40.5%)		
Private Institution	295	14	281		
	(42.8%)	(6.4%)	(59.5%)		

** *p* < 0.01 *** *p* < 0.001.

**Table 2:** Basic service patterns of telemental health service.

	Total	Psychiatrists	Psychologists	χ^2^
	(*n* = 690)	(*n* = 218)	(*n* = 472)	
Teleservice forms (multiple responses allowed)				
Free hotline	380	123	257	0.235
	(55.1%)	(36.4%)	(54.4%)	
Graphic and text	186	118	68	119.498 ***
	(27.0%)	(54.1%)	(14.4%)	
Telephone	303	127	176	26.622 ***
	(43.9%)	(58.3%)	(37.3%)	
Video	455	104	351	47.187 ***
	(65.9%)	(47.7%)	(74.4%)	
Follow-up diagnosis	170	153	17	356.046 ***
	(24.6%)	(70.2%)	(3.6%)	
Prices				
Lower than service on site	260	99	161	28.719 ***
	(37.7%)	(45.4%)	(34.1%)	
Equal to service on site	401	100	301	
	(58.1%)	(45.9%)	(63.8%)	
More than service on site	29	19	10	
	(4.2%)	(8.7%)	(2.1%)	
Age group (multiple responses allowed)				
Child	201	79	122	7.799 **
	(29.1%)	(36.2%)	(25.8%)	
Adolescent	524	168	356	0.220
	(75.9%)	(77.1%)	(75.4%)	
Adult	627	199	428	0.066
	(90.9%)	(91.3%)	(90.7%)	
Old	155	87	68	55.679 ***
	(22.5%)	(39.9%)	(14.4%)	
Treatment genre				
Integrative therapy	234	78	156	64.706 ***
	(33.9%)	(35.8%)	(33.1%)	
Psychoanalytic therapy	170	20	150	
	(24.6%)	(9.2%)	(31.8%)	
Humanistic Therapy	46	7	39	
	(6.7%)	(3.2%)	(8.3%)	
Cognitive Behavioral Therapy	135	67	68	
	(19.6%)	(30.7%)	(14.4%)	
Cognitive Therapy	65	29	36	
	(9.4%)	(13.3%)	(7.6%)	
Behavioral Therapy	7	3	4	
	(1.0%)	(1.4%)	(0.8%)	
Dialectical Behavioral Therapy	4	2	2	
	(0.6%)	(0.9%)	(0.4%)	
Others	29	12	17	
	(4.2%)	(5.5%)	(3.6%)	

** *p* < 0.01 *** *p* < 0.001.

**Table 3:** Ethical service details of telemental health service.

	Total	Psychiatrists	Psychologists	χ^2^
	(*n* = 690)	(*n* = 218)	(*n* = 472)	
Teleservice tools (multiple responses allowed)				
Institution-specific websites or platforms	306	122	184	17.421 ***
	(44.3%)	(56.0%)	(39.0%)	
Collaboration with large platforms	228	117	111	61.281 ***
	(33.0%)	(53.7%)	(23.5%)	
Business conferencing software (e.g., Tencent)	310	56	254	47.676 ***
	(44.9%)	(25.7%)	(53.8%)	
Other communication software (e.g., QQ, WeChat)	321	89	232	4.156 *
	(46.5%)	(40.8%)	(49.2%)	
Email	23	6	17	0.334
	(3.3%)	(2.8%)	(3.6%)	
Telephones	219	81	138	4.316 *
	(31.7%)	(37.2%)	(29.2%)	
Professionals’ location during telemental health services (multiple responses allowed)				
Dedicated space (e.g., specialized spaces in institutions, office space)	485	162	323	2.469
	(70.3%)	(74.3%)	(68.4%)	
Non-dedicated space (e.g., home)	531	161	370	1.731
	(77.0%)	(73.9%)	(78.4%)	
Crisis situation management approach				
The organization can obtain the user’s IP and other location information.	71	20	51	4.592
	(10.3%)	(9.2%)	(10.8%)	
The organization cooperates with the security administration to provide assistance remotely.	141	39	102	
	(20.4%)	(17.9%)	(21.6%)	
The organization has a plan to transfer users offline.	149	42	107	
	(21.6%)	(19.3%)	(22.7%)	
Offline service can only be suggested to the user.	329	117	212	
	(47.7%)	(53.7%)	(44.9%)	
Process documentation				
The host organization keeps all complete electronic records.	235	97	138	28.485 ***
	(34.1%)	(44.5%)	(29.2%)	
The host organization only records simple information, the process is not documented.	88	35	53	
	(12.8%)	(16.1%)	(11.2%)	
Self-documentation of the complete process	197	37	160	
	(28.6%)	(17.0%)	(33.9%)	
Simple self-recording of information, undocumented process	170	49	121	
	(24.6%)	(28.8%)	(25.6%)	

* *p* < 0.05 *** *p* < 0.001.

**Table 4:** Positive response percentages for concerns about telemental health service in different groups.

	*n*	Technical Issues		Confidentiality /Security		Therapeutic Relationship		Standardization		Equipment Cost		Specialized Training	
		%	χ^2^	%	χ^2^	%	χ^2^	%	χ^2^	%	χ^2^	%	χ^2^
Total		63.8	/	62.5	/	54.2	/	43.9	/	10.0	/	23.3	/
Job													
Psychiatrists	218	58.3	4.190 *	69.3	6.289 *	54.1	0.001	58.7	28.352 ***	12.8	2.864	29.8	7.488 **
Psychologists	472	66.3		59.3		54.2		37.1		8.7		20.3	
Gender													
Male	206	69.4	4.057 *	67.5	3.146	55.3	0.153	57.3	21.310 ***	15.0	8.317 **	32.5	13.868 ***
Female	484	61.4		60.3		53.7		38.2		7.9		19.4	
Age													
Below median	359	65.2	0.696	63.2	0.216	55.4	0.400	43.7	0.003	9.7	0.059	23.4	0.000
Above median	330	62.1		61.5		53.0		43.9		10.3		23.3	
Service years						45							
Below median	402	62.9	0.289	60.7	1.283	56.5	1.990	41.0	3.217	9.7	0.095	20.6	3.886 *
Above median	288	64.9		64.9		51.0		47.9		10.4		27.1	
Highest education													
PhD	52	55.8	4.404	67.3	4.299	48.1	7.129	44.2	10.073 *	9.6	1.673	15.4	18.181 ***
Master’s degree	264	65.2		58.7		53.8		39.4		9.1		16.3	
Bachelor’s degree	351	65.0		65.2		57		48.7		10.3		28.8	
Other	23	47.8		52.2		30.4		21.7		17.4		39.1	
Main workplace													
Public institution	395	64.1	0.020	65.3	3.207	55.9	1.135	51.5	22.429 ***	11.1	1.332	28.1	11.741 **
Private institution	295	63.5		58.6		51.9		33.6		8.5		16.9	
	***n***	**Lack of evidence**		**Emergency** **handling**		**Not individually** **suitable**		**Lack of telemental health service knowledge**				**No concerns**	
		**%**	**χ^2^**	**%**	**χ^2^**	**%**	**χ^2^**	**%**		**χ^2^**		**%**	**χ^2^**
Total		21.9	/	71.9	/	4.9	/	8.3		/		4.2	/
Job													
Psychiatrists	218	28.9	9.174 **	75.2	1.765	7.3	3.957 *	10.6		2.204		1.4	6.325 *
Psychologists	472	18.6		70.3		3.8		7.2				5.5	
Gender													
Male	206	28.2	6.756 **	67.5	2.824	6.3	1.199	8.7		0.088		4.4	0.020
Female	484	19.2		73.8		4.3		8.1				4.1	
Age													
Below median	359	21.7	0.016	71.9	0.000	5.6	0.647	7.0		1.693		3.1	2.437
Above median	330	22.1		71.8		4.2		9.7				5.5	
Service years													
Below median	402	21.9	0.000	70.1	1.434	5.0	0.005	9.5		1.805		3.7	0.532
Above median	288	21.9		74.3		4.9		6.6				4.9	
Highest education													
PhD	52	13.5	5.921	65.4	2.924	1.9	3.265	0.0		16.292 **		1.9	9.535 *
Master’s degree	264	19.3		72.0		3.8		5.3				6.8	
Bachelor’s degree	351	25.4		73.5		6.0		10.8				2.3	
Other	23	17.4		60.9		8.7		21.7				8.7	
Main workplace													
Public institution	395	25.6	7.341 **	72.2	0.033	6.6	5.400	10.1		4.244 *		1.8	13.558 ***
Private institution	295	16.9		71.5		2.7		5.8				7.5	

* *p* < 0.05 ** *p* < 0.01 *** *p* < 0.001.

**Table 5:** Positive response percentages for ethical practice in telemental health service in different groups.

	*n*	Received Telemental Health Services Training		Institutional Rules		Knowledge of Association Standards		Knowledge of Laws/Regulations		Informs Benefits/Risks		Informs Data Storage		Informs Data Security		Informs Alternatives		Informs Right to Stop	
		%	χ^2^	%	χ^2^	%	χ^2^	%	χ^2^	%	%	%	χ^2^	%	χ^2^	%	χ^2^	%	χ^2^
Total		69.4		41.4		66.7		58.4		75.4		46.1		71.9		57.5		58.7	
Job																			
Psychiatrists	218	59.6	14.380 ***	38.5	1.117	63.3	1.623	60.1	0.373	77.1	0.497	43.6	0.807	69.3	1.081	61.9	2.514	53.2	3.954 *
Psychologists	472	73.9		42.8		68.2		57.6		74.6		47.2		73.1		55.5		61.2	
Gender																			
Male	206	68.0	0.295	39.8	0.327	69.4	1.000	60.2	0.387	78.6	1.700	47.6	0.261	71.4	0.040	61.7	2.035	52.9	4.051 *
Female	484	70.0		42.1		65.5		57.6		74.0		45.5		72.1		55.8		61.2	
Age																			
Below median	359	70.5	0.425	46.8	9.120 **	64.9	0.992	57.4	0.286	69.9	11.804 **	49.9	4.477 *	72.7	0.273	56.0	0.677	58.8	0.000
Above median	330	68.2		35.5		68.5		59.4		81.2		41.8		70.9		59.1		58.8	
Service years																			
Below median	402	70.6	0.682	42.8	0.709	64.4	2.172	56.7	1.131	69.9	15.474 ***	47.8	1.087	71.9	0.000	54.0	4.985 *	59.5	0.228
Above median	288	67.7		39.6		69.8		60.8		83.0		43.8		71.9		62.5		57.6	
Highest education																			
PhD	52	55.8	7.325	40.4	5.328	59.6	1.733	53.8	5.182	76.9	1.314	42.3	0.532	69.2	0.258	53.8	1.368	61.4	
Master’s degree	264	72.0		36.7		67.0		53.8		73.5		47.3		72.3		60.2		55.8	
Bachelor’s degree	351	70.4		45.6		67.8		62.1		76.1		45.6		71.8		56.1		60.9	
Other	23	56.5		34.8		60.9		65.2		82.6		47.8		73.9		56.5			
Main workplace																		53.9	8.677 **
Public institution	395	68.1	0.757	46.1	8.149	67.3	0.189	62.3	5.704 *	77.7	2.769	48.9	2.861	72.2	0.033	59.7	1.848	58.7	
Private institution	295	71.2		35.3		65.8		53.2		72.2		42.4		71.5		54.6			

* *p* < 0.05 ** *p* < 0.01 *** *p* < 0.001.

**Table 6:** Multiple linear regression analysis of telemental health services provision (R^2^ = 0.326).

	B	S.E.	95% CI		t	*p*
			Lower	Upper		
1 Job	19.748	2.588	14.666	24.830	7.630	0.000
2 Main Workplace	−20.338	2.452	−25.152	−15.523	−8.295	0.000
3 Attitude (ATT)	−0.130	0.510	−1.131	0.872	−0.254	0.799
4 Behavioral intention (BI)	1.314	0.340	0.646	1.981	3.865	0.000
5 Subjective norms (SN)	0.893	0.493	−0.075	1.862	1.811	0.071
6 Perceived ease of use (PEOU)	0.132	0.315	−0.488	0.751	0.417	0.677
7 Perceived behavioral control (PBC)	0.230	0.311	−0.381	0.841	0.738	0.460
8 Perceived usefulness (PU)	0.024	0.383	−0.728	0.776	0.063	0.950

**Table 7:** Multiple linear regression analysis of ethical compliance (R^2^ = 0.105).

	B	S.E.	95% CI		t	*p*
			Lower	Upper		
1 Job	0.578	0.887	−1.165	2.320	0.651	0.515
2 Main Workplace	−0.341	0.841	−1.992	1.309	−0.406	0.685
3 Attitude (ATT)	0.341	0.175	−0.002	0.684	1.950	0.052
4 Behavioral intention (BI)	−0.545	0.117	−0.774	−0.316	−4.674	0.000
5 Subjective norms (SN)	0.401	0.169	0.069	0.733	2.369	0.018
6 Perceived ease of use (PEOU)	−0.075	0.108	−0.287	0.137	−0.692	0.489
7 Perceived behavioral control (PBC)	0.427	0.107	0.217	0.637	4.000	0.000
8 Perceived usefulness (PU)	0.213	0.131	−0.045	0.471	1.621	0.105

## Data Availability

The raw data supporting the conclusions of this article will be made available by the authors without undue reservation.

## Appendix A

Questionnaire content

I. Basic information

1. Your age ____ years old
2. Your gender is: Male/Female
3. Your professional background is: Licensed psychiatrist/Psychotherapist/Counselor
4. Your current main workplace is: Private personal service/Private small services/Private large service/Public hospitals/Military/Campus Health Care/Other public clinics
5. Your highest degree: PhD/Master’s Degree/Bachelor’s Degree/Other
6. The year you worked as a psychological service provider after earning your highest degree was ____

II. Telemental health service patterns

7. You provide an average of ____ hours of telemental health services per week.

Q2 Details of service

(a) You provide online mental health services that include: Free hotline/Graphic and text/Telephone/Video/Follow-up diagnosis
(b) Pricing for online services: Lower than service on site/Equal to service on site/More than service on site
(c) What tools do you currently use to provide online mental health services: Organizations develop dedicated platforms or websites/Collaboration with third-party specialized large platforms (e.g., Good Mood, Simple Minds …)/General business conference software (e.g., Tencent, Attention …)/Other communication software (e.g., QQ, WeChat, Nail …)/Email/Telephone
(d) When providing online services, the locations you typically choose include: Specialized treatment space in an institution/Office space in institutions/Office space in own home/Other private space/Other public space
(e) What age groups do you currently serve: Child/Adolescent/Adult/Old
(f) When providing online mental health services, what is your primary genre of treatment? Integrative therapy/Psychoanalytic therapy/Humanistic Therapy/Cognitive Behavioral Therapy/Cognitive Therapy/Behavioral Therapy/Dialectical Behavioral Therapy/Others
(g) When a user receiving online services is in an emergency situation where there is a risk to themselves or others: The organization can obtain the user’s IP and other location information/The organization cooperates with the security administration to provide assistance remotely/The organization has a plan to transfer users offline/Offline service can only be suggested to the user
(h) How do you record the process of online service? The host organization keeps all complete electronic record/The host organization only records simple information, the process is not documented/Self-documentation of the complete process/Simple self-recording of information, undocumented process

Q3 Concerns (yes/no)

(a) Technical issues
(b) Confidentiality and security
(c) Consultative relationship
(d) Standardization
(e) Equipment costs
(f) Specialized training
(g) Lack of supporting evidence-based research
(h) Inability to handle emergencies
(i) Not suited to individual conditions
(j) No knowledge of online services
(k) No concerns

Q4 Ethical status (yes/no)

(a) Trained in the practical, legal, and ethical aspects of online services.
(b) The organization has specific rules and regulations for online services.
(c) Knowledge of online service standards issued by professional associations.
(d) Knowledge of laws and regulations governing online services.
(e) Inform users of the advantages and disadvantages of online services.
(f) Inform users about how information about online services is stored.
(g) Inform users about the security of information in online services.
(h) Inform the user of alternative ways of providing the service.
(i) Inform users of their right to stop the online service at any time.

III. Attitudes toward telemental health services (1 = complete disagreement, 7 = complete agreement)

1. Using online psychological services in serving and managing visitors is a good idea.
2. Whenever possible, I do not intend to use online psychological services to serve and manage visitors.
3. Respondents want me to provide psychological services remotely/online.
4. Use of online psychological services in serving and managing visitors is beneficial.
5. The operation of online psychological services is not easy for me.
6. I have sufficient resources in providing online psychological services (e.g., I receive training related to online psychological services).
7. I have the ability to use online psychological services in serving and managing my visitors.
8. I have full control over the use of online psychological services.
9. I do not have sufficient knowledge base to provide online psychological services to serve and manage visitors.
10. Using online psychological services does not improve my service and management of my visitors.
11. Supervisor (vs. supervisor) thinks I should not provide psychological services remotely/online.
12. The remote/online approach does not improve the effectiveness of my services and management of visitors.
13. Skilled use of online psychological services is not easy.
14. Use of online psychological services is not useful for the service and management of visitors.
15. Colleagues don’t think I should provide psychological services remotely/online.
16. When I served and managed visitors, I found it easy to access online psychological services.
17. A remote/online approach would make it easier for me to serve and manage my visitors.
18. I found providing psychological services remotely/online easy to use.
19. I would like to provide psychological services to visitors via remote/online as much as possible according to actual needs.
20. Providing psychological services via remote/online makes me uncomfortable.
21. If possible, I would serve my visitors remotely/online as much as possible

IV. Ethical norms (1 = complete disagreement, 7 = complete agreement)

1. I sign a contract with the visitor to establish a service relationship, and the negotiation involving the service relationship in replies to emails, tweets, etc. is also regarded as an agreement or part of it.
2. For visitors who are not comfortable signing a written agreement, I think a verbal agreement can be used to establish a service relationship.
3. When I provide psychological services to minors, I establish a service relationship with their guardians, who also bear the costs, responsibilities and obligations of the visitors.
4. I suspect that a visitor who “claims” to be an adult is a minor and try to determine the age and capacity of the visitor.
5. There were no available follow-up face-to-face service providers and modalities in the visitor’s location, so I informed him/her that it was not appropriate to receive online services.
6. I informed the visitor that although confidentiality measures had been taken, there was still a potential leakage of information with the current technology available.
7. I felt that a visitor’s online service was less than satisfactory and terminated his online psychological service by terminating the appointment.
8. I switched to another room when I was providing online services, even though the other room I switched to was not quiet enough.
9. I do not allow private communication at times other than program setups.
10. Some online seekers of professional services are anonymous, but I ask visitors to ensure that the information related to the counseling issue must be completely truthful.
11. When responding to important emails, etc. involving feedback on results such as advice and guidance to confirm that they have been received, visitors use the auto-reply function and I treat the auto-reply as received.
12. When I suddenly lost power and internet connection in the course of providing online psychological services and could not continue, I rearranged a time with the visitor to do counseling again.
13. I refer to the professional standards of face-to-face psychological services for online psychological services.
14. I evaluate and adjust the program on an ongoing basis, as well as determine the appropriateness of continuing to provide telepsychological services to specific visitors.
15. I provide services 100% in accordance with the previously formulated program.
16. The visitor’s family inquired about the visitor in counseling, and I was forced to inform the family of what the visitor said in counseling.
17. I keep records of “all” electronic communications with visitors, including all work using the Internet and telecommunication technologies; and with their families (including guardians) during the service relationship.
18. I don’t have backups except for accessible forms of recordkeeping.
19. I kept records of visitors for more than three years.
